# Comparison of Muscle Function, Bone Mineral Density and Body Composition of Early Starting and Later Starting Older Masters Athletes

**DOI:** 10.3389/fphys.2019.01050

**Published:** 2019-08-27

**Authors:** Jessica Piasecki, Alex Ireland, Mathew Piasecki, Kevin Deere, Kimberley Hannam, Jonathan Tobias, Jamie S. McPhee

**Affiliations:** ^1^Sport, Health and Performance Enhancement Research Centre, Nottingham Trent University, Nottingham, United Kingdom; ^2^Department of Sport and Exercise Sciences, Musculoskeletal Science and Sports Medicine, Manchester Metropolitan University, Manchester, United Kingdom; ^3^Clinical, Metabolic and Molecular Physiology, MRC-ARUK Centre for Musculoskeletal Ageing Research, National Institute for Health Research (NIHR) Nottingham Biomedical Research Centre, University of Nottingham, Nottingham, United Kingdom; ^4^Musculoskeletal Research Unit, Bristol Medical School, University of Bristol, Bristol, United Kingdom; ^5^Department of Physiology, University of Padua, Padua, Italy

**Keywords:** masters athletes, body composition, bone mineral density, starting age, endurance running

## Abstract

Masters endurance runners can epitomize healthy aging; being reflective of the physiological processes of aging without the compounded effects of inactivity. The primary aim of the present study was to determine, using cross-sectional data, whether individuals taking up training after the age of 50 years can achieve the same level of athletic performance and musculoskeletal characteristics in their older age as those who trained all of their adult lives. A total of 150 master endurance runners [age 68 (5) years; 111 male, 39 female] were divided into early starters (training all of their adulthood) and late starters (started training after age 50 years). A comparative non-athletic group of 59 healthy older adults [age 73 (4) years; 30 female, 29 male] were additionally included for analysis. Training intensity, age-graded performance (AGP) and musculoskeletal assessments were performed. Results showed that there was no difference between athlete groups for training intensity or age-graded performance, despite the 30-year difference in training history. Body fat percentage and leg lean mass did not differ between athlete groups, but were 17% lower and 12% greater, respectively, in athlete groups compared with controls. Power normalized to body mass did not differ between any groups. Spine BMD was lower in late starters than controls, while early starters did not differ from late starters or controls. Hip BMD did not differ between any of the groups. These findings show that the Masters athletes we studied that started intense endurance running after the age of 50 years had lower body fat and higher leg lean mass compared to non-athletes. Body composition and athletic performance of the late starters was very similar to those who trained all of their adult lives.

## Introduction

There are growing numbers of older people training and competing as Masters runners in mass-participation endurance events, such as 5, 10 km, Half Marathon or full Marathons (Stevinson and Hickson, 2014; Lepers and Stapley, 2016). At the highest competitive level, Masters runners train 4–7 times per week at high intensity (Piasecki J. et al., 2018). Their overall lifestyle includes more physical activity than the general population, as they accumulate around 3-fold more low impacts as a result of voluntary movement during general activities, 20-fold more medium impacts and 200-fold more high impacts (measured in units of gravity, *g*) compared with typical older adults from the general population (Deere et al., 2016; Hannam et al., 2016b).

Intense training sustained through middle and into older age helps to maintain low body fat percentage (Piasecki et al., 2019) and, possibly, greater lean mass and skeletal muscle strength into old age. However, the available evidence regarding muscle mass and function of older Masters runners is conflicting. A recent meta-analysis highlighted research gaps due to most studies having low participant numbers with very few female athletes, limited outcome assessments and unclear demographics (Mckendry et al., 2018). For example, two studies (Power et al., 2012; Stenroth et al., 2016) showed higher maximal force or power in Masters endurance runners compared with non-athletic old, but several others showed no difference between Masters endurance runners and controls (Aagaard et al., 2007; Michaelis et al., 2008; Mikkelsen et al., 2013; Couppe et al., 2014; Piasecki et al., 2016; Piasecki J. et al., 2018) or even lower (Sipilä and Suominen, 1991; Sanada et al., 2009) maximal force or power in Masters endurance runners compared with non-athletic old. More recently, Fien et al. (2017) reported high levels of physical function, low body fat, and good health status of Masters athletes, but these characteristics declined with advancing age and because no comparison was made with non-athletic old it is not possible to know if these characteristics were any better than typical healthy old. Differences between studies in the athletic ranking of Masters athletes (e.g., highest achievements), age and years of training are possible explanations for the conflicting reports, but there is very little information available to show how the starting age influences physical performance, muscle mass, and body composition in later life.

In the general population, leg skeletal muscle mass declines progressively from age 30 years at a rate of approximately 8–10% per decade (Lynch et al., 1999; Janssen et al., 2000; Lee et al., 2004; Mitchell et al., 2012; Moore et al., 2014) and maximal force and power decline progressively at approximately 10–12% per decade (Michaelis et al., 2008; Dodds et al., 2014; Bagley et al., 2019). Therefore, by age 50 years non-athletic men and women have typically undergone two decades of progressive muscle declines. If regular endurance running preserves muscle structure and function into older age (Ringsberg et al., 2001; Landi et al., 2018; Shiroma et al., 2018), then life-long athletes should have avoided these age- and lifestyle-dependent declines and should have greater muscle mass and function than those taking up running after the age of 50 years. Although no research is available to support these possible benefits of longer-term training, there is evidence to suggest that starting age is an important factor. A previous study of Masters tennis players found that the upper limb (radius, ulna, and humerus) bone circumference and bone mineral density were greater in those who started training at an early age compared with those starting at an older age (Ireland et al., 2014). However, this study did not examine muscles or bones of the legs that would be exposed to “impacts” during running. More crucially, the positive influence of playing tennis from a young age for arm bones conflicts with other data suggesting that long-term endurance running does not give higher bone mineral density or may even be detrimental for spine, leg or hip bone mineral density (MacKelvie et al., 2000; Wilks et al., 2009a; Piasecki J. et al., 2018).

There is a gap in available knowledge of whether the age at which people take up competitive endurance running influences musculoskeletal structure and function as well as body composition in later life. These questions are important because regular exercise is recommended as the best way to combat age-related declines of the musculoskeletal system (Acsm, 1998; Kusy and Zielinski, 2015; Chief Medical Officer’s Guidelines, 2018; Cruz-Jentoft et al., 2018) and the age at onset of exercise may affect health and performance-related indicators in later life. We therefore aimed to compare body composition, lean mass, maximal power, and hip and spine bone mineral density between Masters endurance runners competing all of their adult lives with those taking up competitive running after the age of 50 years. For reference, a non-athletic healthy older group was also included. It was hypothesized that athletic performance, lean mass, muscle power, and bone mineral density would be greater in trained compared with untrained adults, and greater in those with the longer history of athletic training compared to those starting athletic training after the age of 50 years.

## Materials and Methods

### Study Design

The study was approved by the Regional Ethics Committee (North West England: 14/NW0275) as well the Local Ethics Committee of Manchester Metropolitan University. Written informed consent was obtained from all participants. Masters runners were recruited as part of the United Kingdom-based “VIBE” study (Deere et al., 2016; Hannam et al., 2016b). Male and female athletes were recruited from regional athletics meetings from across the United Kingdom. To be included, they must have been aged ≥59 years at the point of enrollment, had competed in endurance running in the past 12 months at a regional level and free from serious injury for over 2 years. They were invited to attend the research facility at Manchester Metropolitan University (United Kingdom) and were required to avoid training or competing for at least 48 h prior to attendance. The full cohort included 188 Masters Athletes, of which 33 were sprinters (events less than or equal to 400 m) and 150 were endurance runners (events greater than or equal to 800 m). Given the low number of sprinters (when separated to early starters, late starters, men, and women) and the known differences in muscle mass and bone mineral density between sprinters and endurance runners (Gast et al., 2013; Kusy and Zielinski, 2015; Piasecki J. et al., 2018), the sprinters were excluded from further analysis for this particular study. Thus, a total of 150 Masters endurance runners were available for analysis. Mean age-graded performance (AGP) was determined by taking the athlete’s highest ranked performance within the last 2 years and expressing it as a percentage of the world record for that age and distance. AGP ranged from 77–92% across the cohort, indicating a very high level of performance relative to age group records. For example, a 3 h and 30 min marathon for a 70-year-old man gives an age-graded performance of 80%, as described previously (Piasecki J. et al., 2018).

Data for control participants was taken from the MYOAGE study, a European multi-center study of healthy aging and the methodology has been described previously (McPhee et al., 2013). These participants were recruited by focused advertisement in newspapers, the third-generation university and the association of emeriti and universities, thus selecting cognitively active individuals living independently and without mobility impairments. An initial telephone interview was used to screen volunteers, which asked for self-reported number of exercise and social activity sessions per week over the past 3 years. Those who were sedentary defined as not involved in any regular activity sessions, as well as those involved in any form of regular, intense athletic or gym training were excluded from the control group. The controls did not complete accelerometry-based activity monitoring for use in this study. For the purpose of this study the 59 older participants recruited at the Manchester, United Kingdom, site and with complete data including hip and spine bone mineral density measurements were used.

Therefore, the total number of complete datasets available for analysis was 209 and all participants completed measurements at the same laboratory and using the same equipment. Of those, 140 were male and 69 were female. For this study, the athletes were divided into early starter (ES) and late starter (LS) athletes. These groups were based on answers from a questionnaire asking each athlete to self-report the number of hours spent training (0–1, 2–3, 4–7, over 7 per week) during different stages of their adult life (18–29, 30–49 and 50 years and over). Early starter athletes were defined as those that had taken part in competitive endurance running throughout their adult lives, reporting intense training and competition at ages 18–29, 30–49, and 50 years and over. Late starter athletes were defined as those that had taken up intense training and competition after the age of 50 years, with no previous competitive training history. See Table 1 for participant characteristics.

**TABLE 1:** Participant characteristics.

**Variable**	**Group**						**ANCOVA**	**Pairwise comparisons**			***Covariate***	
	**(1) Early starter**		**(2) Late starter**		**(3) Controls**			**1 vs. 2**	**1 vs. 3**	**2 vs. 3**	***Gender***	***Age***
**Sex (n)**	**M (48)**	**F (6)**	**M (63)**	**F (33)**	**M (29)**	**F (30)**						
Adulthood training years	52.3 ± 6.0	47.6 ± 4.2	18.4 ± 5.1	19.4 ± 5.2	N/A	N/A	**>0.0005**	**>0.0005**	**>0.0005**	**>0.0005**	F(1,184) = 0.007, p = 0.934	F(1,184) = 286, p < 0.0005
95% CI	50.7–53.9	43.7–53.2	17.1–19.7	17.2–20.9								
Age (years)^a^	71.3 ± 5.8	66.4 ± 3.0	68.8 ± 5.5	69.6 ± 5.1	74.1 ± 5.7	73.3 ± 4.5	**>0.0005**	0.095	**0.004**	**>0.0005**	F(2,242) = 15.8, p < 0.0005	n/a
95% CI	70.1–73.2	62.9–71.6	67.4–70.1	67.7–71.2	71.9–76.2	71.6–74.9						
Height (cm)	171.2 ± 5.6	164.2 ± 4.5	174.0 ± 6.3	161.6 ± 6.9	172.0 ± 8.7	160.3 ± 5.1	0.411	–	–	–	F(1,242) = 0.142, p = 0.706	(1,242) = 0.455, p = 0.500
95% CI	169.9–173.0	158.4–167.0	172.7–175.6	159.5–164.2	168.6–175.3	158.4–162.3						
Body mass (kg)	68.3 ± 8.7	55.4 ± 4.7	67.5 ± 6.8	56.1 ± 7.8	80.2 ± 16.2	63.1 ± 11.5	**>0.0005**	0.823	**>0.0005**	**>0.0005**	F(1,242) = 84.132, p < 0.0005	F(1,242) = 0.423, p = 0.516
95% CI	67.2–72.4	52.2–60.2	66.8–70.3	54.3–59.7	74.1–86.4	58.8–67.4						
BMI (kg/m^2^)	22.7 ± 4.3	20.5 ± 1.9	22.3 ± 1.9	21.6 ± 2.1	27.1 ± 4.7	24.5 ± 4.2	**>0.0005**	0.864	**>0.0005**	**>0.0005**	F(1,242) = 7.151, p = 0.008	F(1,242) = 0.080, p = 0.778
95% CI	22.1–24.5	20.0–22.4	22.1–23.1	21.1–22.6	25.3–28.8	23.0–26.1						
Accelerometry low impact (0.5–1 g) counts^b^	33529 (21051–46725)	55066 (35520–64137)	44404 (32394–57262)	35637 (23960–52961)			0.089	0.089	–	–	–	F(1,175) = 1.48, p = 0.226
95% CI	28539–40384	19985–56368	39101–50240	32315–46670								
Accelerometry medium impact (1–1.5 g) counts^b^	27565 (10700–50689)	29465 (23566–59147)	34901 (23648–49685)	29868 (20319–40853)			0.80	0.799	–	–	–	F(1,175) = 0.717, p = 0.398
95% CI	20501–36133	9973–42399	28665–39081	24249–40448								
Accelerometry (counts) high impact (>1.5 g) counts^b^	172 (9–1214)	89.6 (50–1572)	221 (32–932)	119 (10–745)			0.35	0.352	–	–	–	F(1,175) = 0.035, p = 0.851
95% CI	386–1217	−269.1–1511	549–3070	−842–5272								
Age graded performance (%)	74.5 ± 1.6	84.3 ± 2.2	77.8 ± 1.3	79.8 ± 1.9			0.29	0.294	–	–	–	F(1,174) = 4.137, p = 0.043
95% CI	72.2–78.3	83.3–92.6	76.1–81.8	76.5–83.8								

*Data are mean ± SD. Age and sex were included as covariates, except where indicated by ^*a*^where sex was the only covariate. ^*b*^Data are presented as median (25th/75th) quartiles. 95% Confidence intervals (95%CI). Bold text is used to highlight statistically significant difference (*p* < 0.05).*

### Questionnaires

Participants provided demographic, general health, lifestyle, and physical activity information by questionnaire [described previously (Hannam et al., 2016b)]. Self-rated health was reported on a scale from very good through to very poor, along with details of any diseases or prescribed medications. Current and history of smoking was collected, including number of cigarettes smoked per normal day, age started smoking and the duration, if ever, of smoking. Typical alcohol consumption was recorded and the type of alcohol. Highest level of education was also recorded. Questionnaire response data has been provided in Supplementary Table S1.

### Accelerometry

Accelerometry data was collected only in the athletes and has been described previously (Deere et al., 2016; Piasecki J. et al., 2018). Each athlete received a GCDC × 16–1c (Gulf Coast Data Concepts, Waveland, MS, United States), which was placed in a Velcro strap and worn tightly around the waist with the accelerometer device placed over their right hip. Each athlete wore a monitor for seven consecutive days, only removing it when showering, bathing, swimming, and sleeping. Each athlete completed a time sheet over the 7-day period to record when the monitor was worn and to indicate any reason why that day was not representative of their usual routine. Accelerometers were configured with standardized settings prior to participant use with a sampling frequency of 50 Hz, a deadband setting of 0.1 g (the threshold which must be exceeded before a recording is made) and a timeout setting of 10 s (meaning that a single sample every 10 s is taken even if the recording is <0.1 g) (Deere et al., 2016). Once the 7 day period of use was completed the participant returned the accelerometer to the research facility by post. The raw accelerometry data was uploaded to a secure shared drive and read into Stata 13 (StataCorp, College Station, TX, United States). A standardized cleaning and processing procedure was used and has been described in detail previously (Deere et al., 2016). In short, the *Y*-axis accelerations dataset was cleaned to remove any movement artifacts and any periods of nil data collection, most likely due to the participant not wearing the accelerometer. Activity data were normalized based on seven valid days of 14 h with ≥10 h recording time. *Y*-axis peaks were calculated based on accelerations that were higher than the previous and subsequent reading and recorded within 14 pre-specified g bands. These were condensed to three impact bands; low (≥0.5 to <1.0 g), medium (≥1.0 to <1.5 g) and higher (≥1.5 g) impact. All *g* values represent *g* over and above 1 *g* from earth’s gravitational force (Hannam et al., 2016a).

### DXA Scans

Standing height was measured to the nearest millimeter and body mass was measured to the nearest 0.1 kg. Whole body, total hip and lumbar spine dual energy X-ray absorptiometry (DXA: Lunar Prodigy Advanced, GE Healthcare, encore version 10.50.086, London, United Kingdom) scans were performed while the participant lay supine wearing a light cotton t-shirt to reduce measurement errors due to clothing absorption. Body composition (fat mass and lean mass) was taken from results of total body scans and regional analysis of legs and arms. Bone mineral density (BMD, g.cm^–2^) was taken from hip and spine scans. All measurements were recorded after manual adjustment of the regions of interest. Repeat total body and hip scans were performed in eight participants within 1 month of the first scan. Using these repeat scans, the short-term error for our laboratory was 2.0% for hip BMD, 0.9% for spine BMD and 0.01% for whole body lean mass.

### Muscle Function

The investigators provided verbal instructions and a physical demonstration of the muscle function tests. Participants were allowed one practice immediately before the actual assessed trials, which acted as a specific warm up and also confirmed that the instructions were understood. In all cases, the muscle function tests were completed between 10 am and 3 pm.

Hand grip strength was measured using the Jamar dynamometer handle (Sammons Preston Inc., Bolingbrook, IL, United States) as previously described (Hannam et al., 2017). The width of the dynamometer was adjusted for each participant separately. Participants were instructed to stand upright with the arm fully extended along the body, maintaining approximately 5 cm gap between the wrist and the hip or upper leg (so that the hand was not rested against the body). Participants were instructed to squeeze against the handle as hard possible for 3 seconds. Grip strength was measured three times and recorded in kilograms to the nearest 0.1 kg. For the purpose of this study, the best of three attempts was included in further analysis.

A Leonardo Jump Mechanography Platform (Leonardo Software version 4.2: Novotiec Medical GmbH, Pforzheim, Germany) was used to assess lower limb muscle power during a countermovement vertical jump, as described previously (Hannam et al., 2017). Results for both absolute (W) and relative (W/kg) power were recorded. Briefly, a two-footed countermovement jump was performed starting with feet approximately 30 cm apart (slightly narrower than shoulder width) and standing upright on the force plates. Force was sampled at 800 Hz. Participants flexed at the knees before extending as forcefully as possible to take off for the jump. Jumps were performed with a trained research assistant in close proximity to intervene in case of a trip or fall. Each participant repeated the jump sequence three times, with approximately 60 seconds rest between efforts. The jump with the highest value for power was used for statistical analysis.

### Statistical Analysis

Statistical analysis was performed using SPSSv21 (IBM, United States). Normality of distribution was assessed using the Shapiro–Wilk test, which showed that all data was normally distributed (presented as mean ± standard deviation) except for accelerometry data (presented as median (25th/75th) quartiles). Two-way ANOVAs (three group and two sex; all *p* > 0.05) showed no group × sex interactions for any of the variables so for this reason, together with the relatively small numbers of females in the ES group, data from both sexes were included within the same analysis. Univariate ANOVA (ANCOVA) analysis was used to identify differences between the three groups (ES, LS, and C) with age and sex added as covariates, to account for the different mean ages and proportions of males and females between groups. Where significant differences were found, Fisher’s Least Significant Difference pairwise comparisons were performed. Differences between groups were considered statistically significant at *p* < 0.05.

As participants included in our study were initially recruited to address other primary research questions, sample sizes for our analyses were fixed by the data already available. Our power to determine small, medium and large effect sizes can be ascertained from the observed effect sizes for the different outcome variables based on the partial eta squared (η^2^*p*), as recommended by Lakens (2013) and O’Keefe (2007) for use with ANOVA analysis including covariates (age and sex). A small effect size was set at η^2^*p* = 0.01, a medium effect size was set at η^2^*p* = 0.06 and a large effect size was set at η^2^*p* = 0.14. The values provided in the Results section show that the study was sufficiently powered to detect even small differences between groups for the main outcome variables of body fat percentage, lean mass, spine BMD, and vertical jump power.

## Results

### Participant Characteristics

Participant characteristics are shown in Table 1 and other health and demographic information has been provided in Supplementary Table S1. The ES athletes had been training for 30 years longer than LS athletes (*p* < 0.0005). Pairwise comparison showed there was no significant difference between ES and LS for AGP (*p* = 0.294). Accelerometry collected over 7 days showed similar impacts in the low, medium, and high *g* bands between ES and LS groups (*p* = 0.089, 0.799, and 0.352, respectively, as shown in Table 1).

**FIGURE 1:**
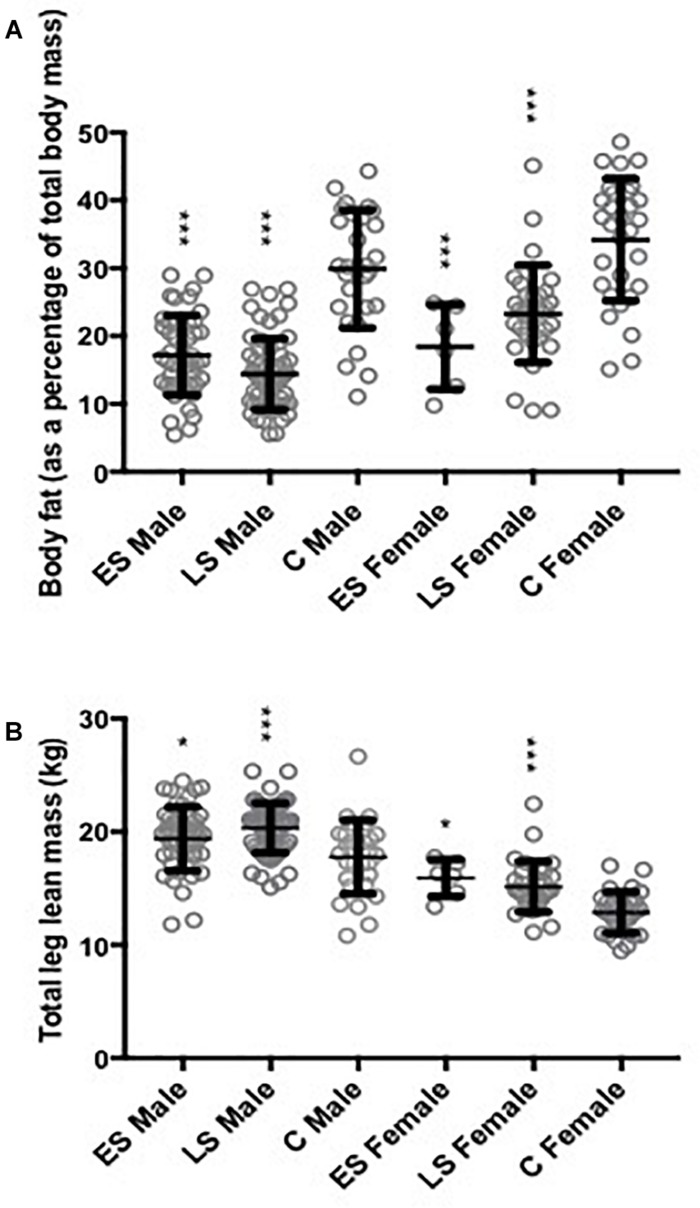
Body fat percentage and leg lean mass. Data shown as mean ± SD and individual data points also displayed as open circles. **(A)** Body fat expressed as a percentage of total body mass, significance assessed when adjusting for Gender [*F*(1,242) = 44.2, *p* < 0.01] and age [*F*(1,242) = 2.07, *p* = 0.15]. **(B)** Leg lean mass significance assessed when adjusting for Gender [*F*(1,242) = 179, *p* < 0.01] and age [*F*(1,242) = 8.40, *p* < 0.01]. ES, Early Starter; LS, Late Starter; C, Controls. ^∗∗∗^ Significantly different to controls at *p* < 0.01 and ^∗^ at *p* < 0.05.

There was no significant difference between groups for height. However, age, body mass, and BMI were significantly different between groups (all *p* < 0.0005). Pairwise comparisons showed that C were older than ES and LS, with no difference between ES and LS groups. Body mass and BMI were similar for the ES and LS athletes, but significantly greater for C compared with ES and LS groups (both *p* < 0.0005). The main reason for greater body mass in C was the higher body fat percentage (Figure 1B).

### Muscle Function and Body Composition

Total body fat percentage differed significantly between groups (*p* < 0.0005; η^2^*p* = 0.380) due to approximately 17% lower values for both athlete groups than C (*p* < 0.01; Figure 1A). Leg lean mass differed significantly between groups (*p* < 0.0005; η^2^*p* = 0.098; Figure 1B), being approximately 12% greater in both athlete groups than C (*p* < 0.003). Appendicular lean mass was also different between groups (*p* = 0.003; η^2^*p* = 0.062) and although values were similar between athlete groups, the LS had a significantly greater lean mass than C (*p* = 0.001). Total body lean mass did not differ significantly between groups (η^2^*p* = 0.028; Table 2).

**TABLE 2:** Musculoskeletal characteristics.

**Variable**	**Group**						**ANCOVA**	**Pairwise comparisons**			***Covariate***	
	**(1) Early starter**		**(2) Late starter**		**(3) Controls**			**1 vs. 2**	**1 vs. 3**	**2 vs. 3**	***Gender***	***Age***
**Sex (n)**	**M (48)**	**F (6)**	**M (63)**	**F (33)**	**M (29)**	**F (30)**						
Total body lean mass (kg)	53.3 ± 8.77	43.1 ± 9.48	54.8 ± 6.1	41.0 ± 9.4	52.3 ± 0.9	38.1 ± 4.3	0.215				F(1,242) = 280, p < 0.0005	F(1,243) = 6.29, p = 0.013
95% CI	51.5–55.1	40.6–45.5	53.6–56.0	39.1–42.8	49.0–55.8	36.4–39.5						
Total body fat mass (kg)	11.9 ± 5.26	10.4 ± 4.02	9.92 ± 4.23	13.2 ± 5.06	24.6 ± 10.3	22.4 ± 9.00	**>0.0005**	0.494	**<0**.**0005**	**<0**.**0005**	F(1,203) = 0.477, p = 0.490	F(1,203) = 0.011, p = 0.917
95% CI	10.3–13.4	6.14–14.6	8.84–11.0	11.4–15.1	20.7–28.9	19.0–25.7						
Appendicular lean mass (kg)	26.0 ± 0.5	19.7 ± 0.7	26.9 ± 0.5	19.0 ± 0.5	23.6 ± 0.8	16.5 ± 0.4	**0.003**	0.084	0.140	**0**.**001**	F(1,242) = 201, p < 0.005	F(1,242) = 10.9, p = 0.001
95% CI	25.0–27.0	19.0–22.2	26.2–28.1	17.8–20.0	22.0–25.4	15.7–17.3						
Spine BMD (g/cm^2^)	1.11 ± 0.02	0.93 ± 0.05	1.08 ± 0.16	0.88 ± 0.03	1.15 ± 0.03	0.95 ± 0.03	**0.004**	0.149	0.087	**0**.**001**	F(1,242) = 71.0, p < 0.005	F(1,242) = 0.77, p = 0.412
95% CI	1.09–1.18	0.87–1.12	1.06–1.13	0.83–0.95	1.08–1.21	0.88–0.99						
Spine BMC (g)	271 ± 8.44	185 ± 18.6	249 ± 8.66	186 ± 1.16	300 ± 12.7	196 ± 11.3	**0.001**	0.091	0.073	**<0**.**0005**	F(1,242) = 60.2, p < 0.0005	F(1,242) = 0.070, p = 0.791
95% CI	256–294	140–303	240–272	173–203	275–331	167–216						
Spine area (cm^2^)	244 ± 4.32	196 ± 10.4	236 ± 3.44	202 ± 3.83	260 ± 6.16	204 ± 8.30	**0.014**	0.403	0.062	**0**.**004**	F(1,242) = 77.2, p < 0.0005	F(1,242) = 0.002, p = 0.966
95% CI	238–253	172–255	231–244	195–210	249–277	182–220						
Hip BMD (g/cm^2^)	1.05 ± 0.02	0.92 ± 0.06	1.02 ± 0.02	0.88 ± 0.02	1.05 ± 0.02	0.88 ± 0.02	0.094				F(1,236) = 58.7, p < 0.0005	F(1,236) = 8.57, p = 0.004
95% CI	1.04–1.12	0.85–1.03	1.00–1.07	0.85–0.93	1.00–1.10	0.81–0.92						
Hip BMC (g)	39.7 ± 0.86	30.6 ± 1.96	37.2 ± 0.89	29.1 ± 0.72	39.0 ± 1.80	28.3 ± 0.94	0.135				F(1,236) = 95.5, p < 0.0005	F(1,236) = 0.372, p = 0.543
95% CI	39.1–42.3	28.5–34.4	36.6–40.1	28.1–30.8	35.3–43.2	25.8–29.9						
Hip area (cm^2^)	37.9 ± 0.53	33.4 ± 0.37	37.0 ± 0.70	33.3 ± 0.40	38.1 ± 063	32.3 ± 0.48	0.617				F(1,236) = 69.6, p < 0.0005	F(1,236) = 6.45, p = 0.014
95% CI	37.1–38.9	31.7–35.8	36.1–38.6	32.3–34.0	37.3–39.7	31.1–33.2						
Maximal grip strength (kg)	36.8 ± 1.2	32.7 ± 3.4	37.3 ± 1.1	35.0 ± 1.9	38.2 ± 1.2	24.4 ± 0.9	0.142	–	–	–	F(1,236) = 32.3, p < 0.0005	F(1,236) = 6.20, p = 0.014
95% CI	35.5–39.8	29.1–37.5	36.3–40.3	31.9–39.1	35.7–40.9	22.3–26.1						
Vertical jump power (W)	2054 ± 75.8	1359 ± 109	2014 ± 74.6	1420 ± 70.5	2191 ± 112.2	1463 ± 89.5	**0.022**	0.504	0.062	**0**.**006**	F(1,238) = 73.2, p < 0.0005	F(1,238) = 28.2, p < 0.0005
95% CI	2016–2368	1169–1838	1967–2282	1314–1612	1997–2481	1243–1646						
Relative vertical jump Power (W/kg)	30.2 ± 1.1	24.5 ± 1.5	30.0 ± 1.1	25.3 ± 1.1	27.5 ± 1.0	23.0 ± 0.9	0.584	–	–	–	F(1,238) = 25.6, p < 0.0005	F(1,238) = 29.8, p < 0.0005
95% CI	29.1–33.5	20.0–32.7	28.8–33.0	23.3–27.8	25.8–29.9	21.2–25.0						

*Data are presented as mean ± SD. Age and sex were included as covariates. Values highlighted in bold text identify significant differences between groups. BMD, Bone mineral density; BMC, Bone mineral content; %Female as displayed in Table 1. 95% Confidence intervals (95%CI).*

Spine BMD (*p* = 0.004; η^2^*p* = 0.053), BMC (*p* = 0.001; η^2^*p* = 0.064), and Area (*p* = 0.014; η^2^*p* = 0.041) were significantly different between groups. There were no differences for these measurements between athlete groups, but LS had lower BMD (*p* = 0.001; η^2^*p* = 0.053), BMC (*p* < 0.0005; η^2^*p* = 0.063), and Area (*p* = 0.004; η^2^*p* = 0.041) compared to C. There was no significant difference in Hip BMD (η^2^*p* = 0.024), BMC (η^2^*p* = 0.020) or Area (η^2^*p* = 0.005) between any of the groups (Table 2).

Maximal grip strength (η^2^*p* = 0.020) and vertical jump power relative to body mass (η^2^*p* = 0.005) did not differ significantly between groups (Table 2). However, vertical jump power in absolute values was different between groups (*p* = 0.020; η^2^*p* = 0.017); values were similar between athlete groups, but controls exhibited a greater power than LS (*p* = 0.006).

## Discussion

The novel contribution of the present study is the focus on the starting age of Masters endurance runners in relation to later life performance and musculoskeletal health indicators. This is supported by the relatively large sample size, depth of physiological profiling and, for the Masters athletes, objective characterization of habitual physical activities. Our findings demonstrate that the men and women of the LS group with no previous history of intense training or competition before the age of 50 years, had by the age of 70 years very similar training intensity, athletic performance, body fat percentage and leg lean mass to athletes of the ES group whom had accumulated 30 extra years of training and competition (Table 2 and Figure 1). Both athlete groups had lower body fat and greater leg lean mass than healthy non-athletic controls, but spine BMD was lower in LS than in C. These findings suggest that starting regular, intense endurance running at, or after, the age of 50 years is not too late to compete at the highest level in Masters endurance running or to significantly delay accumulation of body fat and loss of leg lean mass in older age.

The physical activity levels of ES and LS equates to around 3-fold greater level of low impacts, 20-fold greater level of medium impacts and 200-fold greater level of high impacts per week compared with reference values that were previously reported for older adults from the general population (Hannam et al., 2016b). Past studies have demonstrated both a dose-response and an intensity-dependent response to regular exercise training, as more frequent and higher intensity training confer beneficial health- and performance- adaptations (Swain and Franklin, 2006; Bruce et al., 2008; McPhee et al., 2016). The high impacts and activity levels due to more vigorous intensity movements alongside the associated energy expenditure is the most likely reason why both athlete groups avoided the usual increase in adiposity with advancing older age (Elhakeem et al., 2017).

Appendicular and leg lean mass were similar between athlete groups, but both athlete groups had higher leg lean mass than C (Table 2 and Figure 1). This is in line with two studies (Mikkelsen et al., 2013; Couppe et al., 2014) that reported greater muscle size of Masters endurance athletes than age-matched controls. These past studies (Mikkelsen et al., 2013; Couppe et al., 2014) included only 15 Masters athletes, compared with our much larger sample of 150 endurance runners with detailed activity tracking by accelerometry and confirmation of the high AGP. The prevailing view is that resistance exercise is needed to reduce risk of sarcopenia in old age (Cruz-Jentoft et al., 2018; Lee et al., 2018; Vlietstra et al., 2018). Our results suggest that long term intense endurance running is also effective, and that it does not matter if this activity is taken up after the age of 50 years. However, from the available data we are not able to identify an older age at which benefits of intense endurance running are diminished compared with those training for all of their adult lives.

One of the benefits of having larger muscles is the potential to develop greater muscle force and power. In our study, power measured by vertical jump was not different between athlete groups, but actually lower for LS compared with C. However, this difference between groups disappeared when normalized to total body mass (Table 2). Previous studies also reported similar vertical jump power in Masters endurance runners and age-matched non-athletic individuals (Michaelis et al., 2008). We did not determine the reasons why the larger muscles of endurance runners do not produce greater power, but it is likely related to the characteristic “slow” muscle fiber contractile properties of endurance athletes (Tanaka and Seals, 2008) which makes energy turnover more economical, but gives lower power as the product of force x velocity of contractions (Michaelis et al., 2008). We did not measure knee extensor maximal force, but the available evidence is conflicting over the possibility that Master endurance runners have greater maximal force, mainly due to heterogeneity in study populations and several studies with low sample size (Mckendry et al., 2018).

The LS athletes had lower spine BMD, BMC, and Area than C, raising the possibility that starting regular intense endurance running after age 50 years may be detrimental to spine bone health. This may seem counter-intuitive, as it is proposed that regular exercise with high impacts can improve bone mineral density and bone strength (Ireland et al., 2011, 2013) as high muscular forces stimulate osteogenic responses (Frost, 1987a, b). However, this previous literature is primarily based on observations of long limb bones (Wilks et al., 2009a, b; Ireland et al., 2011, 2014). For example, previous studies showed that the limb bone circumference of Masters athletes (33–94 years old) was greater than that of age-matched sedentary controls (Wilks et al., 2009b). These beneficial effects may be limited to limb bones, younger ages or participation in sprint or power activities (Warden et al., 2007, 2014; Wilks et al., 2009b; Ireland et al., 2014). Previous research has also demonstrated an inverse association between the amount of low-impact physical activity and hip and spine BMD (Johansson et al., 2015; Hannam et al., 2016b; Piasecki J. et al., 2018). Although the explanation is lacking for why completing lots of low impact activity may be detrimental for bones, it is well known that bone responses depend on the type of physical activity being performed (Nichols et al., 2003; Velez et al., 2008; Wilks et al., 2009a; Piasecki J. et al., 2018; Pollock et al., 2018). For instance, sprinting is associated with greater hip, spine and tibial BMD compared to endurance running or non-athlete controls (Wilks et al., 2009a; Piasecki J. et al., 2018). Although not measured in the present study, Vitamin D and calcium intake are also associated with skeletal health in older age (Cashman, 2007). In particular, Vitamin D deficiency and low calcium intake can lead to low bone mineral density and they are used as supplements to combat osteoporosis (Grados et al., 2003a, b). Therefore, any interventions later in life to improve bone health may need to include nutritional supplementation, and also consider sprint or jumping activities in addition to regular prolonged endurance running.

Overall, our findings build upon available evidence that short-term exercise can improve some features of musculoskeletal health in middle- and older-age (McPhee et al., 2016) and have highlighted possible benefits of very long-term exercise (Velez et al., 2008; Wilks et al., 2009a, b; Nowak et al., 2010; Ireland et al., 2015; Mckendry et al., 2018), although these benefits may not be present for lumbar spine bones. The novel contributions of the present study describing the very long-term intense training and the focus on starting age are important because starting at a later age risks the possibility that irreversible age-related declines have already occurred and would limit adaptations that improve health and performance. For example, age-related reduction of maximal heart rate limits cardiac output and therefore peak aerobic capacity. Within the musculoskeletal system the skeletal muscle mass, strength and power decline from the fourth decade of life (Lynch et al., 1999; Janssen et al., 2000; Silva et al., 2010; Reid and Fielding, 2012; Moore et al., 2014; Pantoja et al., 2016; Fien et al., 2017; Bagley et al., 2019). The extent to which these changes are related to irreversible reductions of muscle fiber numbers (Lexell et al., 1988; McPhee et al., 2018) and motor units (Tomlinson and Irving, 1977; Piasecki M. et al., 2018; Piasecki et al., 2019) remains unknown. In this respect, the findings of the present study are encouraging. The implication based on observations made from the participants in our study, is that starting intense training in middle age, approximately 20 years after musculoskeletal declines are detectable in the general population, is not a disadvantage compared to training throughout adult life when it comes to maintaining leg muscle mass, preventing accumulation of excessive fat mass and for athletic performance in older age.

The findings of this study may inform practitioners when recommending physical activity for older adults aiming to reduce fat mass, gain leg lean mass or improve bone mineral density. The new information showing that long-term endurance training is associated with greater leg lean mass should be considered by policy makers as an alternative, or addition, to resistance exercise to combat sarcopenia. One note of caution, however, is that the lower bone mineral density of the spine in LS may increase the risk of late life bone injuries.

### Study Limitations

The grouping of ES and LS has relied on self-reports and the cross-sectional study design prevents any causal relationships from being established. Our sample size of 209 provided sufficient power to detect even small differences in means between comparison groups, but we cannot rule out the possibility that there may be very small differences in outcomes between the ES and LS groups which we were unable to detect. There is also a risk of differences in data handling and screening of bone results (e.g., for spondylosis) when comparing the results of the Masters athletes with those of the controls, where data were collected approximately 5 years apart. However, care was taken to follow exactly the same protocols as far as possible and the assessments were made in the same laboratory using the same equipment. The values for body composition and lean mass of the controls are within the ranges previously published for non-athletic but otherwise healthy older adults from larger, multi-center studies (Bijlsma et al., 2013; Bauer et al., 2015; Coulson et al., 2017; Verlaan et al., 2017). Nevertheless, interpretation of outcome comparisons for the control group is limited because they did not complete the same physical activity assessments as the Masters athletes. The athletes were recruited based on their athletic performance over the previous 2 years. Within this time period there may have been fluctuations in activity levels due to injury or illness which were not captured within this study. Future studies could shorten the time period taken into account when recruiting the athletes or more carefully consider their time spent inactive. More detailed information about youth physical activity may also add further insights. A B-PAQ questionnaire (Weeks and Beck, 2008) may be used in future for this purpose. Studies of Masters athletes carry a possible bias because any individuals developing injury or disease may cease competing and would not be available for recruitment. Masters athletes tend to be better educated, of higher socio-economic status and with fewer diseases than the general population (Supplementary Table S1), so the results of such studies may not be generalized to the wider population. Finally, we cannot rule out possible differences in energy intake or nutritional status, particularly of Vitamin D and calcium that influence musculoskeletal health, between athletes and controls because this was not measured in our study.

## Conclusion

The Masters athletes within our sample taking up intense endurance running after the age of 50 years had lower body fat and higher leg lean mass than non-athletes by the age of 70 years and the values for body composition and athletic performance of the late starters were very similar to those of people whom had trained all of their adult lives.

## Ethics Statement

The study was conducted in accordance with the Declaration of Helsinki and approved by the University Research Ethics Committee and the National Research Ethics Committee Northwest (14/NW0275). All participants provided written informed consent.

## Author Contributions

All authors contributed to the data collection, analyses, and wrote the manuscript. JP led the write up and directed the analysis of data.

## Conflict of Interest Statement

The authors declare that the research was conducted in the absence of any commercial or financial relationships that could be construed as a potential conflict of interest.
