# Supplementary material for: Comparison of Muscle Function, Bone Mineral Density and Body Composition of Early Starting and Later Starting Older Masters Athletes
Source: Front Physiol. 2019 Aug 27;10:1050. doi: 10.3389/fphys.2019.01050 (PMC6719569; doi:10.3389/fphys.2019.01050)
Supplement: Supplementary file 1 [file Table_1.docx]

| Variable | Coding | ES | LS | C |
| --- | --- | --- | --- | --- |
| Marital Status (%) | 1. Single and never married 2. Married and living with husband/wife 3. Married and separated form husband/wife 4. Divorced 5. Widowed 6. Registered/civil partnership 7. Cohabiting | 3  78  3  8  4  0  4 | 0  66  6  11  14  0  3 | 13  65  6  0  16  0  0 |
| Education level (%) | 1-Educated to maximum college diploma or A-level  2- Educated to minimum university degree or professional qualification | 41  59 | 42  58 | 82  18 |
| Current Health (%) | 1. Very good 2. Good 3. Fair 4. Poor | 61  35  4  0 | 69  30  1  0 | 10  81  7  2 |
| Diseases (%) | 1. Chronic non- specific lung disease 2. Diabetes 3. Cancer 4. Arthritis 5. Thyroid issues | 11  4  4  21  4 | 1  0  5  17  2 | 0  4  6  12  13 |
| Taking prescribed medications (%) | 1. Yes 2. No | 85  15 | 44  56 | 58  42 |
| Median number of medications |  | 1.0 ± 0.0-2.0 | 0.0 ± 0.0-1.3 | 2.0 ± 0.0-3.0 |
| Current Smokers | 1. Yes 2. No | 19  81 | 10  90 | 3  97 |
| Drink Alcohol | 1. Yes 2. No | 85  15 | 87  13 | 72  28 |

**Supplemental Table 1. Participant Health and Demographic Characteristics.**
